# Case of a Solution of Continuity in the Parietal Bones, Not Consequent to External Injury

**Published:** 1810-05

**Authors:** George Hargrove

**Affiliations:** Member of the Royal College of Surgeons, London, and Assistant Surgeon in the Royal Artillery


### Medical and Physical Journal.

VOL. XXIII.] MAY, 1810. [NO. 135.

*Printed for R. PHILLIPS, by W. Thorne, Red Lion Court, Fleet Street, London.*

### *Case of a Solution of Continuity in the Parietal Bones, not consequent to external Injury;* by GEORGE HARGROVE, Member of the Royal College of Surgeons, London, and Assistant Surgeon in the Royal Artillery.

WILLIAM SMYTH, a Sergeant in the Artillery, aged thirty-eight years, is a married man, and the father of two or three fine children; he is apparently delicate, but this I attribute to the existence of the affection; in other respects he enjoys a sound and healthy constitution. After investigating the history of his health from the earliest years, exclusive of his appearance at present, I have been unable to ascertain any symptom that would indicate a tendency to the scrophulous habit, nor have I any reason to believe he has suffered from a syphilitic affection, to which I could impute the production of the extraordinary effects now apparent.

About seven years since he was first attacked with a wandering pain of his head, which, agreeably to his account, set in with a degree of violence scarcely possible to conceive. It was accompanied by a large, soft, and puffy tumour, which subsided and rose again at different intervals, shifting its situation from one temple to the other, and from the forehead to the summit and back part of the head. He describes it as having been of a stinging and lancinating nature; and what appears remarkable, he found no alleviation from his sufferings, except when he stooped forward, resting his head on his hands; and when in this incumbent position, a sudden accession of lethargic stupor uniformly produced a suspension from pain. At times, however, a slight remission occurred; and during one, of a more permanent description, he was ordered to proceed on duty to Malta, where he quickly regained his health and spirits, and pursued his military avocations for two years, with the same effect he had done before he had been seized with the affection of his head. He was now ordered to return to England, in the usual routine of duty, and had only been about two months at Portsmouth, when the pain and tumours came on in a most distressing manner. After a stay of some months at the latter place, during which he had a misera-ble existence, he was ordered to Guernsey, while in one of his temporary remissions of pain, and shortly recover-ed, so as to enjoy perfect health in this island for five years, at the expiration of which time he was again or-dered home, and the complaint returned with such seve-rity, as to be unequalled by any former attack.

A circumstance particularly worthy of observation is, that since the first accession of those extraordinary pains, he had never been in better health than when on foreign service, nor did he ever suffer more than when, for any length of time, in his native country, which the preced-ing paragraph sufficiently corroborates.

About the beginning of June, 1809, the pains and tu-mours became unusually troublesome, so much so, that if he wore his regimental cap even for a few minutes, one continued tumefaction surrounded the edge of it. He in-formed me, that shortly before this period he fell sudden-ly, from an universal dizziness that affected him, but re-ceived no hurt or accident by the fall, nor any that he could call to his recollection from his earliest years. In June, a painful phlegmonous tumour, about the size of a small walnut, arose over the middle of the sagittal suture, which suppurated, and burst with a very small open-ing; this gave exit to a trivial discharge, but was not suc-ceeded by an abatement of his pain. It was now deemed expedient to dilate the opening, when a large collection of pus, of an apparently good consistence, was discharg-ed. In a few days after, slight exfoliations were observed to come off piecemeal, to the great surprise of the medi-cal gentlemen attending him, until at length the dura ma-ter was laid bare. This membrane was found to have re-ceded from its attachment to the cranium for the extent of half an inch around the aperture.

The man now first began to express his sense of amend-ment, and shortly after mentioned that he felt complete relief from his pains. This opening is situated precisely over the great longitudinal sinus, and is now about an inch in circumference. Another matter of surprise is, that it has been discharging ever since, without remission, ge-nerally to the extent of about two wine glasses in twentyfour four hours, but at present is reduced to one. At this moment I observe the motion of the brain pumping off (if I may use the phrase) a quantity of purulent matter of a yellow colour and thin consistence. I have observed an osseous matter filling up the aperture, but it is perforated, and seems to give way for the discharge. The dura mater does not appear disposed to throw up that peculiar fungus so often observed after applications of the trephine, nor to suffer in the least from its frequent unavoidable exposure; the edges of the integuments around the opening are inverted, and do not seem disposed to close over the aperture. During the exfoliation of the bones, the man sunk into a state of debility from the quantity of pus hourly secreted and evacuated; he got better, however, from the use of bark and exercise; these, indeed, have been the only means he has made use of for the recovery of his health. He does not suffer the least pain or inconvenience from the opening, or from the application of a sponge or cloth; and it is simply defended from the influence of the atmosphere by a compress of lint and a piece of flannel. This man is an out-patient, and generally visits the Hospital twice or thrice a week.

I should have mentioned, that another tumour lately appeared over the frontal bone; it has been opened, and discharged a quantity of thick pus. The part is not painful, and the chances are, I fear, that it will terminate in a state perhaps similar to the other, although it has not been attended with inflammatory symptoms to any thing near the same extent.

REMARKS.

This curious affection, in my mind, bears a more striking analogy to Necrosis, than to any other disease I have heard of; indeed, many of the symptoms the man labored under, were those of that disease; but still the consideration of other attendant circumstances induces me to adopt a different opinion, although those circumstances may ultimately prove destitute of a source sufficiently plausible to account for them. I am led to imagine that the effect produced has been the operation of Nature, for the purpose of giving exit to some offending quality that existed, I suspect, within the cranium, which was evidently productive of the most serious consequences, considerably affecting the functions of the brain. This was manifest from the man's sufferings prior to the formation of the opening in the bones. But here I am at a loss to account

 for for the absence of other concomitant symptoms usually present on such occasions; however, the continual state of distracting pain the man had been in; the complete cessation of it on the opening being formed by *spontaneous* exfoliations; and his being totally free from this pain for those last eight or nine months *while in England*, during which time it has been throwing off such an immense quantity of purulent matter; together with the remarkable fact of the pain being changed for a state of stupor and sleep, as long as the head was placed in that position most favourable for the diffusion of a preternatural fluid, if such did exist,—are severally, events that strongly incline me to adhere to the last Supposition, in preference to any other which my short experience could adduce, or my imagination is capable of suggesting.

The case, however involved in obscurity, is an instance in addition to numberless others, elucidating the wonderful powers of Nature for the completion of her designs: for, we observe that a considerable portion of the highly requisite defence and support of that important organ the brain, is, in toto, removed, and even from its peculiarly strong adhesion to the dura mater along the course of the suture, and over the longitudinal sinus, most certainly for some great cause, which the instantaneous change in the man's health plainly evinces; thus producing an effect which has occupied the attention of authors and practitioners since the existence of the records of Surgery, when the result of external injury.

Should I, at a future period, have an opportunity of ascertaining the final issue of this case, I shall feel happy in forwarding the communication.

*Royal Artillery Hospital, Woolwich,*
*March* 18, 1810.

